# Simultaneous quantile regression and determinants of under-five severe chronic malnutrition in Ghana

**DOI:** 10.1186/s12889-020-08782-7

**Published:** 2020-05-07

**Authors:** Justice Moses K. Aheto

**Affiliations:** Department of Biostatistics, School of Public Health, College of Health Sciences, University of Ghana, P. O. Box LG13, Legon-Accra, Ghana

**Keywords:** Quantile regression model, Height-for-age, Stunting, Child malnutrition, Risk factors, Malnutrition determinants, Developing countries, Sub-Saharan Africa, Ghana

## Abstract

**Background:**

Under-five malnutrition is a major public health issue contributing to mortality and morbidity, especially in developing countries like Ghana where the rates remain unacceptably high. Identification of critical risk factors of under-five malnutrition using appropriate and advanced statistical methods can help formulate appropriate health programmes and policies aimed at achieving the United Nations SDG Goal 2 target 2. This study attempts to develop a simultaneous quantile regression, an in-depth statistical model to identify critical risk factors of under-five severe chronic malnutrition (severe stunting).

**Methods:**

Based on the nationally representative data from the 2014 Ghana Demographic and Health Survey, height-for-age z-score (HAZ) was estimated. Multivariable simultaneous quantile regression modelling was employed to identify critical risk factors for severe stunting based on HAZ (a measure of chronic malnutrition in populations). Quantiles of HAZ with focus on severe stunting were modelled and the impact of the risk factors determined. Significant test of the difference between slopes at different selected quantiles of severe stunting and other quantiles were performed. A quantile regression plots of slopes were developed to visually examine the impact of the risk factors across these quantiles.

**Results:**

Data on a total of 2716 children were analysed out of which 144 (5.3%) were severely stunted. The models identified child level factors such as type of birth, sex, age, place of delivery and size at birth as significant risk factors of under-five severe stunting. Maternal and household level factors identified as significant predictors of under-five severe stunting were maternal age and education, maternal national health insurance status, household wealth status, and number of children under-five in households. Highly significant differences exist in the slopes between 0.1 and 0.9 quantiles. The quantile regression plots for the selected quantiles from 0.1 to 0.9 showed substantial differences in the impact of the covariates across the quantiles of HAZ considered.

**Conclusion:**

Critical risk factors that can aid formulation of child nutrition and health policies and interventions that will improve child nutritional outcomes and survival were identified. Modelling under-five severe stunting using multivariable simultaneous quantile regression models could be beneficial to addressing the under-five severe stunting.

## Background

Even though malnutrition can be prevented and treated, it remains one of the biggest threats to public health globally. Malnutrition among children aged below 5 years is a major public health issue, especially in developing countries like Ghana. Childhood malnutrition has adverse effects on the survival, growth and cognitive development of the child throughout their life span and its total negative repercussions for the nation such as low productivity and high cost of public health expenditure. Thus, it is a threat to human and economic progress of nations globally. This limits children to realise their full mental and physical potentials in life [1–6]. Reducing malnutrition prevalence in children aged below 5 years is central to the United Nations Sustainable Development Goals 2 target 2. Globally, 22.2% of children aged below 5 years in 2017 were stunted (chronic malnutrition). In 2017, 39% of stunted children lived in Africa, and across all the regions, Africa is the only region that witnessed an increase in the number of stunted children in 2017. Sub-Saharan Africa is one of the regions with the highest levels of child malnutrition globally [7, 8]. In Ghana, the prevalence of stunting, underweight and wasting were 19, 11 and 5% respectively in 2014 [9], suggesting that the malnutrition prevalence, especially stunting in the country is still high and unacceptable in relation to the efforts in achieving the SDG 2 [10] target 2.

Studies have shown that child malnutrition increases the risk of child mortality. Globally, about 45% of under-five deaths were attributable to malnutrition in 2017 and accounted for over 50% of under-five deaths in developing countries. In Ghana, malnutrition accounted for 40% of childhood mortality [5, 11–13].

There have been several national policies and interventions in Ghana to improve child nutrition and health outcomes. Some of these include the National Nutrition Policy 2014–2017, Child Health Policy 2007–2015, National Health Insurance, and Community-based Health Planning and Services policy [9, 14, 15]. Despite these efforts, under-five malnutrition, especially stunting remains high in Ghana.

Majority of the studies [1, 4, 16–19] conducted to examine risk factors of under-five malnutrition in Ghana used statistical models which might not be optimal to inform targeted nutrition policies and interventions because they could not give much information about the underlying associations, not robust to statistical outliers and lacks flexibility in analysing the determinants of nutritional status. For example, modelling the mean as in the ordinary linear regression models could miss critical aspects of the relationship that may exist between the nutritional status and its determinants, especially in the presence of skewed data as is usually the case with anthropometric data [20–24].

Identification of critical risk factors of under-five malnutrition using appropriate and advanced statistical modelling technique was considered in this study to help formulate appropriate nutrition and health programmes and policies aimed at achieving the United Nations Sustainable Development Goal (SDG) 2 [10] target 2. Specifically, the study attempts to develop a multivariable simultaneous quantile regression model as an in-depth statistical model to identify critical risk factors of under-five severe stunting (severe chronic malnutrition) to aid targeted policy and intervention strategies aimed at reducing the prevalence.

## Methods

### Data

This work was based on the nationally representative data from the 2014 Ghana Demographic and Health Survey (GDHS) [9] which is cross-sectional study in nature. The MEASURE DHS Program provided the dataset for this work and is freely available online at the MEASURE DHS Program website [25] upon request. Ghana is one of the countries participating in the collection of data on population, anthropometry and health indicators like maternal and child health, family planning methods and use, household socioeconomic status, and nutritional status of children and women. Nationally representative samples of 12,832 households from 427 clusters were selected based on a two-stage sample design. A total of 11,835 eligible households were interviewed and data collected on 9396 women aged 15–49 years. Data were also collected on 4388 men aged 15–59 years. Detailed survey methods are published elsewhere [9]. Data on 2716 children aged below 5 years with complete information on anthropometric measurements for height-for-age z-scores and covariates considered for the study were generated from the women dataset.

### Outcome variable

The outcome variable of interest in the study is height-for-age z-score (HAZ) measured on a continuous scale. The HAZ was estimated based on WHO growth standards [26, 27]. Multivariable simultaneous quantile regression modelling was employed to identify critical risk factors for severe stunting based on HAZ (a measure of chronic malnutrition in populations). The HAZ was modelled based on quantiles. The primary focus of this study is to model severe form of stunting (HAZ < -3) which corresponded to quantiles between 0.01 and 0.20 inclusive according to the WHO Growth Standards.

### Covariates

This study considered several covariates based on literature on factors influencing under-five malnutrition, especially in developing countries [1, 4, 28–31]. Factors considered at the individual child level include type of birth, sex, age, diarrhoeal episode, place of delivery and size at birth. Maternal and household level factors considered include maternal age, number of children < 5 years in households, maternal national health insurance status and education, and household wealth status.

### Statistical analyses

Descriptive analyses were conducted to summarize the characteristics of the sample using frequencies and their associated percentages. Simultaneous quantile regression modelling approach [20–22, 32] was employed to investigate determinants of severe stunting corresponding to the quantiles of [0.01, 0.20]. The models were also extended beyond severe stunting to capture the full distribution of HAZ. Thus, to examine the effects of the risk factors at different points of the conditional distribution of (HAZ). Generally, the quantile regression is used to describe conditional quantiles of the outcome variable in relation to covariates rather than modelling the mean. Thus, quantile regression modelling is a statistical procedure used to model quantiles (percentiles) within a regression framework. It has been shown extensively in the literature that modelling anthropometric measurements, especially nutritional status through quantile regression approach is more appropriate than modelling the mean (mean regression). Thus, quantile regression model is robust non-normal data and statistical outliers, gives much more information about the underlying associations and provides flexibility in analysing the determinants of nutritional status corresponding to quantiles of interest either in the lower tail (e.g. 0.10), the median (0.50) or the upper tail (e.g. 0.90) of the distribution rather than investigating only the determinants of the mean distribution [20–23]. It is obvious that distributions may not only vary by their means but also by their upper or lower tails so modelling the mean as in linear regression models could miss critical aspects of the relationship that may exist between the response variable and its determinants, notable in the presence of skewed data as is usually the case with medical and health data. However, quantile regression is rarely used in the medical and health data modelling despite its advantages over linear regression, especially when modelling anthropometric outcomes.

Let *Y* be the outcome of interest (i.e. HAZ in this case) and *X* a vector of observed covariates. We can model the ***τ*** quantile of *Y* conditional on *X* = *x* using the quantile regression model given as

1 $$ {Q}_{y_i\mid {x}_i}\left(\boldsymbol{\tau} |{\boldsymbol{x}}_{\boldsymbol{i}}\right)={\boldsymbol{x}}_i^T{\boldsymbol{\beta}}_{\boldsymbol{\tau}} $$

where, ${Q}_{y_i\mid {x}_i}\left(\tau |{x}_i\right)$ is the conditional *τ*^th^ quantile outcome given ***x***_***i***_**,***τ* ∈ (0, 1) is the *τ*^th^ quantile of the outcome variable (HAZ). For example, ***τ*** = [0.01, 0.20] for severe stunting regression and *τ* =0.5 for median HAZ regression. ***x***_***i***_ = (*x*_*i*1_, *x*_*i*2_, …, *x*_*ip*_)^*T*^ is the vector of covariates for each individual ***i***, and ***β***_***τ***_ = (*β*_*τ*0_, *β*_*τ*1_, *β*_*τ*2_, …, *β*_*τp*_)^*T*^ is the vector of (p + 1) regression coefficients at a known ***τ***. The formulation in (1) permits modelling of two or more quantiles of HAZ simultaneously while adjusting for the observed covariates.

To achieve the primary objective, multivariable simultaneous quantile regression models were fitted to the quantiles of 0.10 and 0.20, both measuring severe form of stunting. The lower tail of 0.10 was used instead of the lower tail in [0.01, 0.20] for severe stunting to increase the number of observations falling within the 0.10 quantile of HAZ. In meeting the secondary objective, the impact of covariates on the quantiles of 0.1, 0.2, 0.3, 0.4, 0.5, 0.6, 0.7, 0.8, and 0.9 were examined using multivariable simultaneous quantile regression model. Results from the model were used to develop plots of covariate effects on the quantiles of HAZ used in the model to permit visual examination of the covariate effects on each quantile. This is to provide a more complete picture of the distribution, but not only for severe stunting which can be used to identify more vulnerable groups and to target interventions to these groups. All analyses were done in R version 3.5.2 [33]. The quantile regression analyses were implemented using R package quantreg [34]. To declare statistical significance, a *p*-value below 0.05 was used.

## Results

### Background characteristics

Data on a total of 2716 children were analysed out of which 144 (5.3%) were severely stunted. 123 (4.5%) of them were products of multiple births, majority (51.8%) of them are males while 1499 (55.2%) of them were aged 25–59 months. 331 (12.2%) and 414 (15.2%) of them had diarrhoea and fever respectively. Majority (68.7%) of them were delivered at health facility, 443 (16.3%) were born small at birth, 1334 (49.1%) of the children had mothers aged 26–35 years. Majority of the children belonged to households with more than 3 children below 5 years, 1904 (70.1%) belonged to mothers with health insurance, 1613 (59.4%) of them are been currently breastfed, majority (53.8%) of them belonged to poor households and 972 (35.8%) belonged to mothers with no formal education (Table 1).

**Table 1:** Summary of selected background characteristics (*n* = 2716)

Variables	***n***(%)
**Severely stunted**	
No	2572 (94.7)
Yes	144 (5.3)
**Type of birth**	
Single	2593 (95.5)
Multiple	123 (4.5)
**Sex**	
Male	1406 (51.8)
Female	1310 (48.2)
**Age**	
0–24 months	1217 (44.8)
25–59 months	1499 (55.2)
**Had diarrhoea**	
No	2385 (87.8)
Yes	331 (12.2)
**Had fever**	
No	2302 (84.8)
Yes	414 (15.2)
**Place of delivery**	
Health facility	1866 (68.7)
Home	850 (31.3)
**Size at birth**	
Large/average	2273 (83.7)
Small	443 (16.3)
< 26 years	715 (26.3)
26–35 years	1334 (49.1)
> 35 years	667 (24.6)
**Number of children < 5 years**	
**1–3**	1477 (54.4)
**> 3**	1239 (45.6)
**Health insurance status**	
Had no health insurance	812 (29.9)
Had health insurance	1904 (70.1)
**Currently breastfeeding**	
No	1103 (40.6)
Yes	1613 (59.4)
**Wealth status**	
Average/ rich	1255 (46.2)
Poor	1461 (53.8)
**Maternal educational level**	
Primary/higher	1744 (64.2)
No formal education	972 (35.8)

### Determinants of severe stunting for quantiles of 0.1 and 0.2

The models identified child level variables such as type of birth, sex, age, place of delivery and size at birth as significant risk factors of under-five severe stunting. Maternal and household level variables identified as significant predictors of under-five severe stunting were maternal age and education, maternal national health insurance status, household wealth status, and number of children under-five in household.

Children who are products of multiple births, older ages of children, been delivered at home, born small at birth, increase in number of children < 5 years in households, belonging to poor households and having mother with no formal education are associated with increased risk of severe stunting. Being a female child, increase in maternal age, and mothers who had health insurance was associated with decreased risk of severe stunting (Table 2). The study did not observe any significant differences in slopes across the 0.10 and 0.20 quantiles as all *p*-values, including the overall p-value exceeded 0.05. Thus, the effects of covariates (or slopes) on the 0.10 and 0.20 quantiles of HAZ are the same.

**Table 2:** Results of multivariable simultaneous quantile regression analysis for under-five child severe stunting

Variables	Quantile = 0.1			Quantile = 0.2		
	β	95% CI	***p***-value	β	95% CI	***p***-value
**Child characteristics**						
**Type of birth**						
Single	ref			ref		
Multiple	−0.849	(−1.202, − 0.53)	< 0.001***	− 0.847	(− 1.044, − 0.55)	< 0.001***
**Sex**						
Male	ref			ref		
Female	0.172	(0.052, 0.267)	0.013*	0.153	(0.062, 0.227)	0.01*
**Age (months)**	−0.015	(− 0.021, − 0.009)	< 0.001***	− 0.015	(− 0.018, − 0.011)	< 0.001***
**Had diarrhoea**						
No	ref			ref		
Yes	−0.149	(− 0.345, − 0.029)	0.196	− 0.16	(− 0.353, − 0.012)	0.08
**Place of delivery**						
Health facility	ref			ref		
Home	−0.199	(− 0.358, − 0.046)	0.02*	−0.179	(− 0.298, − 0.096)	0.007**
**Size at birth**						
Large/average	ref			ref		
Small	−0.339	(− 0.537, − 0.18)	< 0.001***	−0.373	(− 0.485, − 0.269)	< 0.001***
**Maternal/Household**						
**Maternal age**	0.016	(0.006, 0.027)	0.021*	0.024	(0.016, 0.033)	< 0.001***
**Children < 5 years**	− 0.192	(− 0.352, − 0.034)	0.038*	−0.224	(− 0.343, − 0.13)	0.005**
**Health insurance**						
Had no health insurance	ref			ref		
Had health insurance	0.237	(0.131, 0.386)	0.002**	0.304	(0.21, 0.388)	< 0.001***
**Wealth status**						
Average/ rich	ref			ref		
Poor	−0.259	(−0.398, −0.12)	0.001**	−0.281	(− 0.371, − 0.183)	< 0.001***
**Maternal education**						
Primary/higher	ref			ref		
No formal education	−0.358	(− 0.556, − 0.223)	< 0.001***	−0.256	(− 0.349, − 0.124)	0.001**

β: coefficient of parameter. CI: confidence interval. *: *p*-value < 0.05. **: *p*-value< 0.01. ***: *p*-value < 0.001. ref.: reference category

**Note:** The DHS categorises the wealth index as poorest, poor, middle, richer and richest. However, in this work, we combined poorest and poorer as poor. Also, middle, richer and richest were combined as average/rich. Similar recoding was done for size at birth, place of delivery and maternal education

### Effects of covariates on quantiles of HAZ ranging from 0.10 to 0.90

Table 3 revealed that significant differences exist in slopes between the quantiles of 0.10 (q0.10) and 0.90 (q0.90) of HAZ. The slopes of type of birth (q0.10: **β =** − 0.849, 95% CI: − 1.202, − 0.53; q0.90: **β =** − 0.334, 95% CI: − 0.457, 0.150; *p*-value = 0.021), child age (q0.10: **β =** − 0.015, 95% CI: − 0.021, − 0.009; q0.90: **β =** − 0.023, 95% CI: − 0.027, − 0.020; *p*-value = 0.006), maternal age (q0.10: **β =** 0.016, 95% CI: 0.006, 0.027; q0.90: **β =** 0.035, 95% CI: 0.024, 0.045; *p*-value = 0.048) and education (q0.10: **β =** − 0.358, 95% CI: − 0.556, − 0.223; q0.90: **β =** − 0.052, 95% CI: − 0.208, − 0.116; p-value = 0.008) varied significantly across the 0.10 and 0.90 quantiles of HAZ. The overall p-value of 0.004 suggests strong differences in slopes between 0.10 and 0.90 quantiles of HAZ.

**Table 3:** Results of multivariable simultaneous quantile regression analysis for under-five child severe stunting

Variables	Quantile = 0.1			Quantile = 0.9		
	β	95% CI	***p***-value	β	95% CI	***p***-value
**Child characteristics**						
**Type of birth**						
Single	ref			ref		
Multiple	−0.849	(−1.202, − 0.53)	< 0.001***	− 0.334	(− 0.457, 0.150)	0.044*
**Sex**						
Male	ref			ref		
Female	0.172	(0.052, 0.267)	0.013*	0.049	(−0.085, 0.168)	0.505
**Age (months)**	−0.015	(−0.021, − 0.009)	< 0.001***	−0.023	(− 0.027, − 0.020)	< 0.001***
**Had diarrhoea**						
No	ref			ref		
Yes	−0.149	(− 0.345, − 0.029)	0.196	− 0.249	(− 0.418, − 0.044)	0.013*
**Place of delivery**						
Health facility	ref			ref		
Home	−0.199	(− 0.358, − 0.046)	0.02*	− 0.100	(− 0.241, 0.068)	0.283
**Size at birth**						
Large/average	ref			ref		
Small	−0.339	(−0.537, − 0.18)	< 0.001***	− 0.397	(− 0.530, − 0.233)	< 0.001***
**Maternal/Household**						
**Maternal age**	0.016	(0.006, 0.027)	0.021*	0.035	(0.024, 0.045)	< 0.001***
**Children < 5 years**	−0.192	(−0.352, − 0.034)	0.038*	−0.338	(− 0.459, − 0.193)	0.001**
**Health insurance**						
Had no health insurance	ref			ref		
Had health insurance	0.237	(0.131, 0.386)	0.002**	0.294	(0.164, 0.422)	< 0.001***
**Wealth status**						
Average/ rich	ref			ref		
Poor	−0.259	(−0.398, −0.12)	0.001**	−0.250	(− 0.390, − 0.124)	0.004**
**Maternal education**						
Primary/higher	ref			ref		
No formal education	−0.358	(−0.556, − 0.223)	< 0.001***	−0.052	(− 0.208, − 0.116)	0.591

β: coefficient of parameter. CI: confidence interval. *: *p*-value < 0.05. **: *p*-value< 0.01. ***: *p*-value < 0.001. ref.: reference category

**Note:** The DHS categorises the wealth index as poorest, poor, middle, richer and richest. However, in this work, we combined poorest and poorer as poor. Also, middle, richer and richest were combined as average/rich. Similar recoding was done for size at birth, place of delivery and maternal education

### Quantile plots of covariate effects for the ranges of 0.10 to 0.90 quantiles of HAZ

**Fig. 1:**
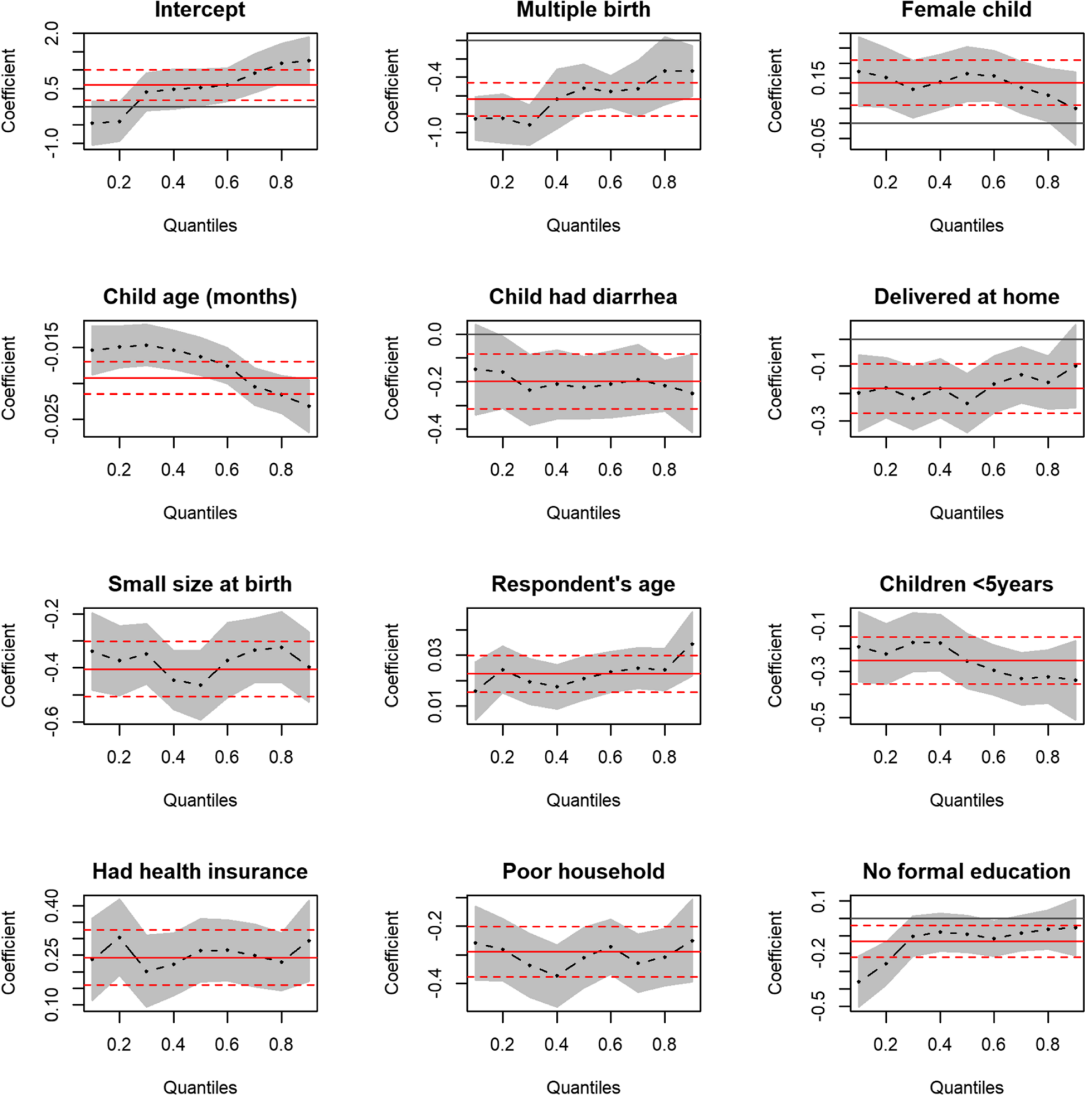
Plot of covariate effects on quantiles from multivariable simultaneous quantile regression (black dots) and their associated 95% confidence interval (gray shaded regions). Note: The quantiles used are 0.1, 0.2, 0.3, 0.4, 0.5, 0.6, 0.7, 0.8, and 0.9 represented by the dots (.) in the plots. The solid red lines are the ordinary least square regression lines with their 95% confidence intervals (dashed red lines)

**Fig. 2:**
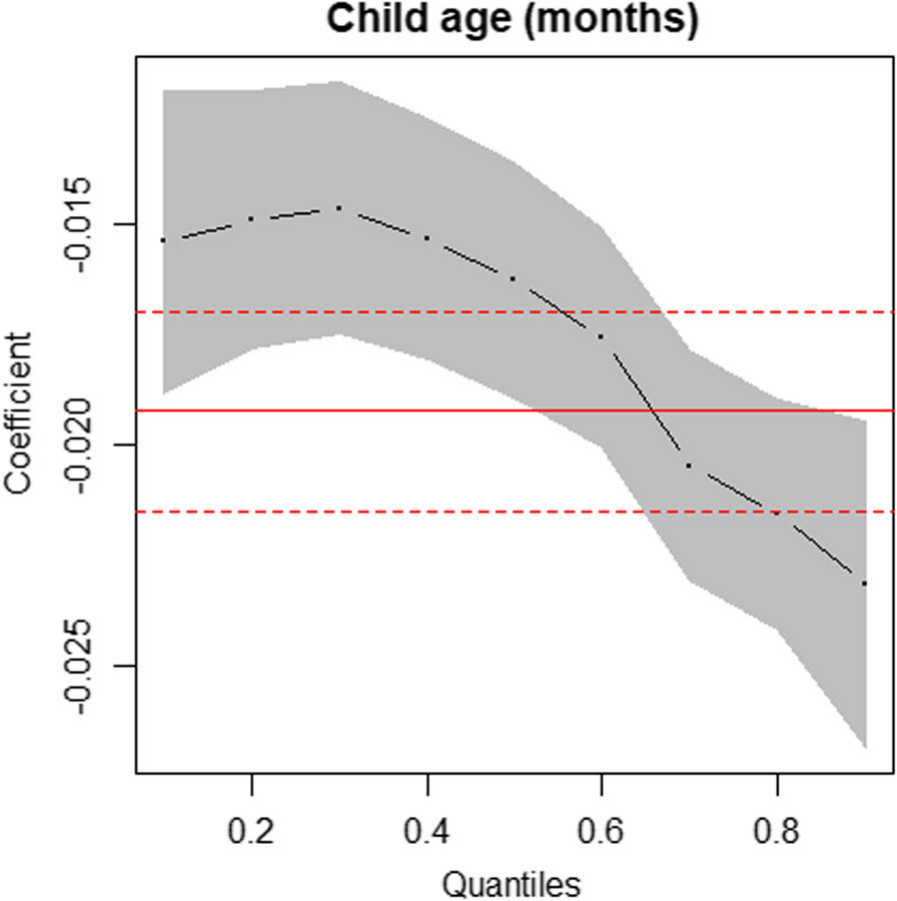
Plot of child’s age effect on quantiles from multivariable simultaneous quantile regression (black dots) and their associated 95% confidence interval (gray shaded regions). Note: The quantiles used are 0.1, 0.2, 0.3, 0.4, 0.5, 0.6, 0.7, 0.8, and 0.9 represented by the dots (.) in the plots. The solid red lines are the ordinary least square regression lines with their 95% confidence intervals (dashed red lines)

**Fig. 3:**
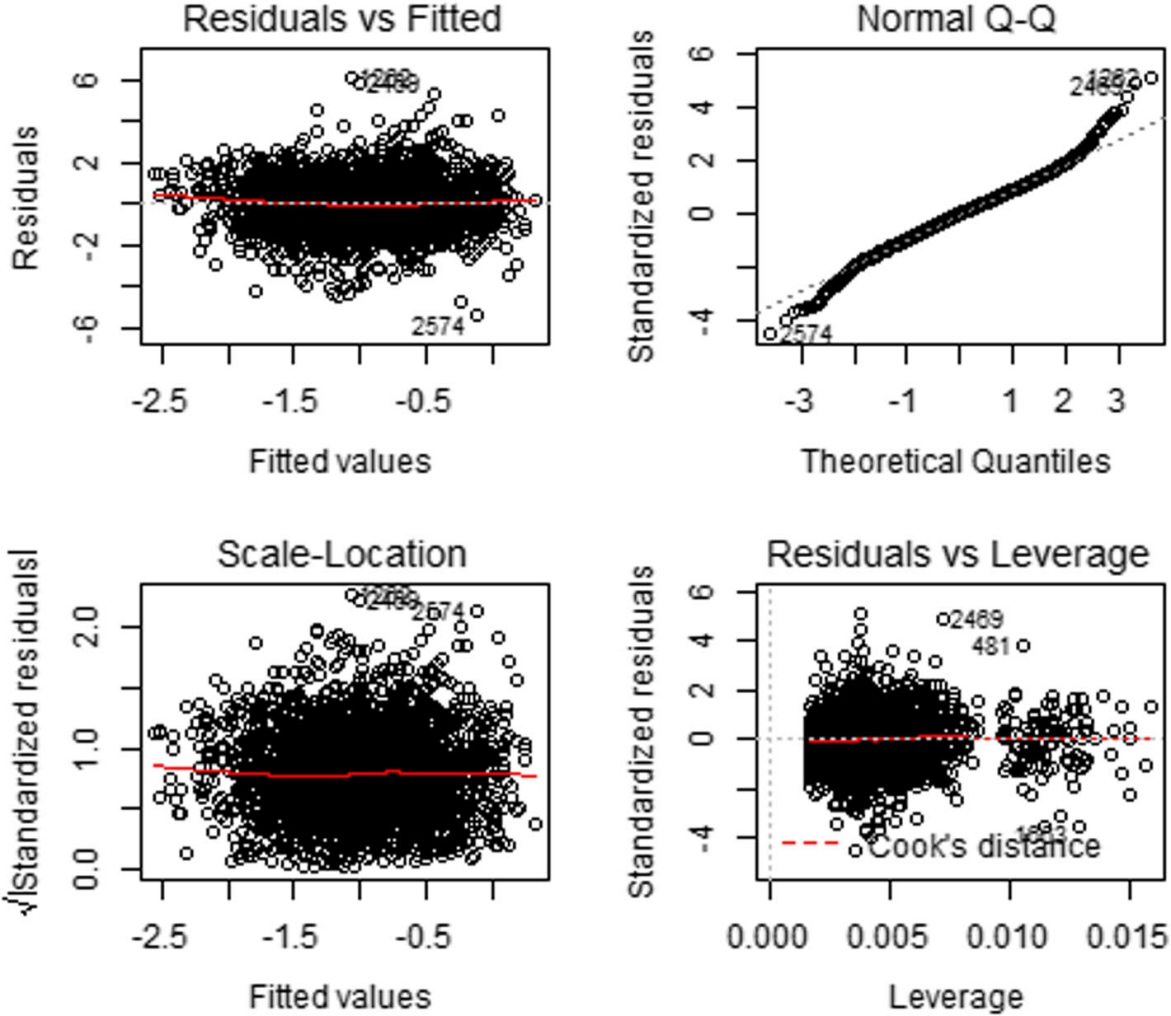
Residual diagnostic plots for the linear regression model for HAZ

The results presented in Fig. 1 is the plot of covariate effects on quantiles of HAZ from multivariable simultaneous quantile regression (black dots) and their associated 95% confidence interval (gray shaded regions). The solid red lines in the plots are the ordinary least square (linear) regression lines with their 95% confidence intervals (dashed red lines). Also, presented in Table 4 is the result from the linear regression model. In Fig. 2, an attempt was made to provide a larger scale figure for child’s age covariate for better examination of the effects on the varying quantiles of HAZ. Figure 1 revealed that some covariates (e.g. multiple birth and no formal education) had minimal effects at the lower quantiles of HAZ and greater effects at higher quantiles of HAZ while others (e.g. child age and number of children< 5 years) had greater effects at the lower quantiles of HAZ and minimal effects at upper quantiles of HAZ. The quantile regression provided a good fit to the data, especially at the lower or/and upper tails of the distribution for covariates like child age, multiple births, and maternal education than the ordinary least squares regression (i.e. solid and dotted red lines in Figs. 1 and 3) which is expected as discussed under the statistical analyses section. Using the same HAZ outcome variable and covariates used in the quantile regression to fit the standard linear regression, the study observed departure from normality and constant variance assumptions. In addition, there was presence of outliers (Fig. 3). A formal test conducted to examine constant variance assumption using Breusch-Pagan test confirmed the violation of the assumption (BP = 43.99, df = 11, *P*-value< 0.001). Thus, the linear regression model for HAZ could not provide a good fit to the data. Diagnostic plots and the formal test conducted suggest that a non-parametric model could be preferred to a parametric model.

**Table 4:** Results of multivariable linear regression analysis for under-five child height-for-age z-scores

Variables	β	95% CI	***p***-value
**Child characteristics**			
**Type of birth`**			
Single	ref		
Multiple	−0.640	(−0.858, − 0.422)	< 0.001***
**Sex**			
Male	ref		
Female	0.136	(0.046, 0.225)	0.013**
**Age (months)**	−0.019	(−0.022, − 0.017)	< 0.001***
**Had diarrhoea**			
No	ref		
Yes	−0.199	(− 0.337, − 0.061)	0.005**
**Place of delivery**			
Health facility	ref		
Home	−0.182	(−0.290, − 0.074)	0.001**
**Size at birth**			
Large/average	ref		
Small	−0.405	(− 0.527, − 0.283)	< 0.001***
**Maternal/Household**			
**Maternal age**	0.023	(0.014, 0.031)	< 0.001***
**Children < 5 years**	−0.251	(− 0.373, − 0.129)	< 0.001***
**Health insurance**			
Had no health insurance	ref		
Had health insurance	0.243	(0.143, 0.343)	< 0.001***
**Wealth status**			
Average/ rich	ref		
Poor	−0.289	(−0.394, − 0.184)	< 0.001***
**Maternal education**			
Primary/higher	ref		
No formal education	−0.131	(− 0.237, − 0.024)	0.016***

β: coefficient of parameter. CI: confidence interval. *: *p*-value < 0.05. **: *p*-value< 0.01. ***: *p*-value < 0.001. ref.: reference category

**Note:** The DHS categorises the wealth index as poorest, poor, middle, richer and richest. However, in this work, we combined poorest and poorer as poor. Also, middle, richer and richest were combined as average/rich. Similar recoding was done for size at birth, place of delivery and maternal education

## Discussion

The study aims to develop a novel quantile regression models to identify critical risk factors of under-five severe stunting (severe chronic malnutrition). The study also attempts to examine the effects of risk factors on different quantiles of HAZ to inform appropriate malnutrition policies and intervention strategies.

Analyses were conducted on 2716 children out of which 144 (5.3%) were identified as severely stunted. Critical risk factors associated with severe stunting were type of birth, sex, age, place of delivery, size at birth, maternal age and education, maternal national health insurance status, household wealth status, and number of children under-five in households.

The use of simultaneous quantile regression to model severe stunting (i.e. 0.10 and 0.20 quantiles of HAZ) as well as ranges of 0.10 to 0.90 quantiles of HAZ as opposed to ordinary least squares regression is of paramount interest in this study. The simultaneous quantile regression modelling approach employed in this study provided a richer characterization of the data, thereby revealing the effect of a covariate on the entire distribution of HAZ, making it possible to identify the more vulnerable groups and to formulate more effective interventions to these groups.

The results have shown that quantile regression models provided much more information about the underlying associations better than the ordinary least squares regression, suggesting that the conditional distributions of HAZ did not only differ by their means but also by their lower and upper tails as reported in previous studies [20–24]. Thus, the ordinary least squares regression missed critical aspects of the associations that exist between the conditional distributions of HAZ and its determinants as shown in Fig. 1. For example, the impact of child age and the number of children below 5 years in households on HAZ was higher at the lower quantiles of HAZ and lower at the higher quantiles of HAZ. This finding suggests that children at the lower tail of the HAZ distribution who are more likely to be severely stunted benefit more from interventions aimed at addressing issues surrounding child age and number of children below 5 years in households than those in the upper end of the distribution. Thus, child age and number of children below 5 years in households interventions to address under-five severe stunting could have more impacts on children who are at higher risk of severe stunting. Also, the impact of multiple births and mothers with no formal education on HAZ was lesser at the lower tail but higher at the upper tail, suggesting that children at the lower tail of the HAZ distribution who are more likely to be severely stunted benefit less from interventions aimed at addressing issues surrounding multiple births and improvement in maternal education than those in the upper end of the distribution.

The study broadly supports earlier studies that examined determinants of under-five malnutrition in developing countries. Multiple births, older children, been delivered at home, born small at birth, increase in number of children aged < 5 years in households, belonging to poor households, and having mothers with no formal education increased the risk of under-five severe stunting. Factors that were protective of under-five severe stunting include female child, older ages of mothers, and having a mother who had health insurance [1, 4, 28, 35–42].

Children belonging to mothers with no formal education had increased risk of severe stunting compared to their counterparts with primary education or higher. Increase in the level of maternal education has been shown to be protective of child malnutrition [1, 4, 40, 43, 44]. The plausible explanation could be that mothers who had better education may have knowledge about better childcare and childhood feeding practices, better health seeking behaviour, and judicious in the management of the limited household resources. Well educated mothers are expected to be more conscious about their children nutrition and health [19, 36–38, 40, 43, 45].

The finding that children from poor households are more likely to be severely stunted compared to their counterparts from average/rich households is consistent with previous studies [1, 36, 38, 40]. This could be as a result of low quality and inadequate food consumption, exposure to infections and lack of access to health services commonly associated with poor households. Thus, the effect of poverty could be manifested through lower purchasing power, low literacy, and food insecurity resulting in poor nutritional outcomes among poor households [1, 16, 36, 37, 43].

This study supports previous studies that reported that children from Mothers who had health insurance had lower risk of severe stunting compare to those who had mothers with no health insurance [1, 44, 46]. The possible explanation could be that having national health insurance could propel mothers to seek better health care for themselves and their children, visit health facilities and this is expected to result in improved health outcomes for children and their mothers [1, 47, 48]. The association observed among children from older mothers and the decrease in severe stunting suggests encouraging adult motherhood as young mothers are less likely to have good knowledge on appropriate health care and feeding practices of their children. Also, young mothers need adequate nutrition for their optimal growth and that of their children but this is not always the case as mothers have to share the little portion of food with their babies [40, 49–51]. An increase in the likelihood of severe stunting found among children from households with higher number of children below 5 years could be due to increased competition for household food with a high number of people and limited resources to respond to health needs of household members. Households with more children are generally socioeconomically disadvantaged coupled with poor quality of life, suggesting the need for family planning and improvement in socioeconomic conditions of households [18, 19, 40, 41, 52, 53].

The finding that the risk of severe stunting was higher among children who were born multiple compared to those born singletons is consistent with previous studies [1, 28, 42]. This could be attributable to premature births, low birthweight, cerebral palsy or competition for nutritional intake commonly observed among children who are products of multiple births [1, 28, 42, 54] which has the tendency of limiting child growth. Consistent with previous studies, female children had decreased risk of severe stunting compared to their male counterparts [28, 38, 42]. Generally, female children are less likely to be influenced by environmental adverse effects than male children. Thus, gender inequality in childhood malnutrition is more likely to be observed in hostile environments such as diseases and exposure to environmental pollutants. Apart from epidemiological evidence that male children are biologically more vulnerable to morbidity [55, 56], gender-based cultural practices and perceptions could influence the behavioural patterns of care-givers in which female children are given preferential feeding treatments [57, 58].

Increase in the likelihood of severe stunting observed among older ages of children in this study supports previous studies [1, 4, 28, 40, 42, 59]. A plausible explanation could be a deficit in timely and adequate complementary feeding and presence of progressive childhood diseases [1, 4, 28, 59]. The finding that the children who were born small at birth had higher likelihood of severe stunting is consistent with previous studies. Size of child at birth is a proxy for child birthweight and that low birthweight is a risk factor for under-five child malnutrition [1, 60–62]. Children born at non-health facility had higher likelihood of severe stunting compared to their counterparts delivered at health facilities, a finding which is consistent with previous studies [35, 60, 63, 64]. A plausible reason could be that mothers who delivered at non-health facilities were not assisted by a skilled provider [35].

The strengths of this study include the fact that it utilised data from a nationally representative population-based survey which is globally respected for its sound survey methods and sound data quality on children, their households and communities in which they live. The large samples drawn nationwide permits generalization of findings to the population of children under-five in Ghana and that of children from other similar populations globally. The study also used a novel simultaneous quantile regression approach, permitting the study of covariate effects across different quantiles of HAZ. Thus, providing much more information about the underlying associations between HAZ and the risk factors which could not have been possible using ordinary linear regression approach. Despite these strengths, the study has a limitation. The survey employed cross-sectional study design which could not establish cause and effect relationship between severe stunting and risk factors considered. Also, it is very difficult measuring income of households in developing countries like Ghana. As a result, an asset-based wealth index commonly used as a proxy for household income was used in this study.

One of the major public health issues in Ghana is under-five child malnutrition with the prevalence of stunting been the highest. The findings in the present study provided vital and current information on critical risk factors for under-five severe stunting that can be used by policymakers and health practitioners for better understanding of under-five severe stunting and its prevention and management. The study highlights the need for improvement in maternal education and general socioeconomic conditions of households, intensification of family planning education and its services, encouraging adult motherhood and targeting nutrition and health policies and intervention strategies at teenage mothers and their children, educating and encouraging women to delivery at health facilities and supporting women and their children with free national health insurance premiums can be helpful in reducing the risk of severe stunting and its associated morbidity and mortality.

## Conclusion

This study developed a novel simultaneous quantile regression model which identified child, maternal and household level variables as critical risk factors of under-five severe stunting. The critical risk factors identified can aid formulation of child nutrition and health policies and interventions that will improve child nutritional outcomes and survival. Improvement in maternal education and household socioeconomic status, making national health insurance freely and easily accessible to mothers and their children, promoting adult motherhood and delivery at health facilities, and targeting households with children born multiple and small size at birth as primary interventions are warranted as part of the overall strategy to ameliorate the nutritional status of children. The study recommends the use of quantile regression modelling techniques in analysing determinants of malnutrition in populations to provide a richer characterization of the data which will permit identification of more vulnerable groups and to formulate more effective nutrition interventions to these groups. Using fully Bayesian quantile regression approach to model severe stunting among children aged below 5 years could be explored as a further research.

## Data Availability

Data supporting the conclusions of this article are freely available at URL: https://dhsprogram.com/data/available-datasets.cfm upon making request to the DHS Program.

## Abbreviations

GDHS: Ghana Demographic and Health Survey
SDG: Sustainable Development Goal
